# A Handbook of Post Mortem Examinations and of Morbid Anatomy

**Published:** 1873-05

**Authors:** 


### Books Reviewed.

*A Hand-Book of Post-Mortem Examinations and of Morbid Anatomy.* By Francis Delafield, M. D. New York: William Wood & Co., 1872. Buffalo: H. H. Otis.

A work of this character has been for some time one of the unsupplied necessities of the profession. Physicians who have found it necessary to make post-mortem examinations, either in search of pathological facts or in behalf of the authorities, have often found their labor lost on account of a lack of knowledge of the steps to be taken in making a proper post-mortem examination. The carelessness with which some of these examinations are made, and the total disregard of pathological teachings which characterizes the testimony presented to many of our coroners' juries, tend to convince those who are cognizant of the facts that there is a great need of reform. But as long as the professson continue to "go it blind," as it were, just so long will "heart disease" and "apoplexy" continue to constitute the verdicts rendered by our coroners' juries. We know of no one who is better calculated to write a book of the character of the one under consideration than Dr. Delafield. His large experience in post-mortem examinations and in the department of morbid anatomy, has borne fruit of which the profession may well be proud. The work may be looked upon as a guide, whose reliability need not be questioned, and whose devotion to facts, rather than theories, will render its teachings a thousand-fold more valuable.

The work is divided into four parts: Part first treats of the method of making post-mortem examinations; Part second of the lesions to be observed in different portions of the body; Part third considers the lesions found in cases of general disease, of poisoning, and of violent death; Part fourth is devoted to a consideration of tumors.

Each division of the work is well written, and the remarks of the writer are based upon established facts, it being his desire to present a book to the profession which would serve as a guide in making post-mortem examinations rather than a work on pathology. The methods of making post-mortem examinations are well given, and no physician who follows the teaching of the author need go far astray in his examination. This portion of the work occupies about forty pages. The succeeding two hundred and sixty pages are taken up by the second division. The several lesions observed in the different organs of the body are all carefully given. This will be found to be an interesting and valuable portion of the work. The remaining pages — about seventy in number — comprise Parts three and four, and are written in the general good style which pervades the whole work. The classification and names of tumors given are a modification of those proposed by Virchow; the modifications, however, are slight. The book is one which all physicians should read, and which should be especially studied by those in the habit of making post-mortem examinations; it is to this class that it will be found to be of especial value. We cannot too highly recommend it to the attention of the profession.

